# A Sampling-Based Algorithm with the Metropolis Acceptance Criterion for Robot Motion Planning

**DOI:** 10.3390/s22239203

**Published:** 2022-11-26

**Authors:** Yiyang Liu, Yang Zhao, Shuaihua Yan, Chunhe Song, Fei Li

**Affiliations:** 1 Key Laboratory of Networked Control Systems, Chinese Academy of Sciences, Shenyang 110016, China; 2 Shenyang Institute of Automation, Chinese Academy of Sciences, Shenyang 110016, China; 3 Institutes for Robotics and Intelligent Manufacturing, Chinese Academy of Sciences, Shenyang 110169, China; 4 Kunshan Intelligent Equipment Research Institute, Kunshan 215300, China; 5 School of Automation and Electrical Engineering, Shenyang Ligong University, Shenyang 110159, China; 6 School of Computer Science and Technology, University of Chinese Academy of Sciences, Beijing 100049, China; 7 College of Information Science and Engineering, Northeastern University, Shenyang 110819, China

**Keywords:** motion planning, sampling-based algorithms, RRT, Metropolis acceptance criterion, asymptotic optimality

## Abstract

Motion planning is one of the important research topics of robotics. As an improvement of Rapidly exploring Random Tree (RRT), the RRT* motion planning algorithm is widely used because of its asymptotic optimality. However, the running time of RRT* increases rapidly with the number of potential path vertices, resulting in slow convergence or even an inability to converge, which seriously reduces the performance and practical value of RRT*. To solve this issue, this paper proposes a two-phase motion planning algorithm named Metropolis RRT* (M-RRT*) based on the Metropolis acceptance criterion. First, to efficiently obtain the initial path and start the optimal path search phase earlier, an asymptotic vertex acceptance criterion is defined in the initial path estimation phase of M-RRT*. Second, to improve the convergence rate of the algorithm, a nonlinear dynamic vertex acceptance criterion is defined in the optimal path search phase, which preferentially accepts vertices that may improve the current path. The effectiveness of M-RRT* is verified by comparing it with existing algorithms through the simulation results in three test environments.

## 1. Introduction

Robotics is evolving rapidly and has dramatically improved the efficiency of industrial production and the convenience of people’s lives. Motion planning is indispensable in robotics, which requires finding a feasible path from the initial state to the target state subject to obstacle avoidance constraints. Nowadays, motion planning has been widely applied to various robots, including but not limited to industrial robots [1,2], free-floating space robots [3], rescue robots [4], medical robots [5], and autonomous vehicles [6].

According to the research order and fundamental principles, various motion planning algorithms can be mainly divided into four categories: bionic algorithms, artificial potential field methods, grid-based searches, and sampling-based algorithms [7]. The ant colony algorithm [8], one of the representative bionic algorithms, draws a lesson from the behavior of ants exploring paths to find food, showing strong robustness. However, it has the problems of slow convergence and a poor quality of the solution when dealing with large-scale problems [9]. The artificial potential field (APF) [10] method assumes a gravitational force of the goal state and repulsive forces of the obstacles. By calculating their resultant force, the following motion state of the moving object can be determined. Though APF has a simple structure and a small amount of computation, it is faced with the disadvantages of a local minimum value, large path oscillations, and complex path searches between similar obstacles [11]. The grid-based searches represented by A* [12,13] map motion planning problems into graphs and solve them in discrete state spaces. Although A* can return to the path with a minimal cost, the computation time and storage space of the data grow exponentially as the dimension of the state space increases.

Compared with grid-based searches, sampling-based algorithms avoid discretizing the state space in motion planning and efficiently perform in high-dimensional state spaces, such as Rapidly exploring Random Tree (RRT) [14] and Probabilistic RoadMap (PRM) [15]. Since RRT-based algorithms have the characteristics of a high search efficiency and low resource consumption, they have been frequently used to solve manipulators, autonomous surface vehicles, and other robot motion planning problems [16,17,18]. Furthermore, many variants of RRT have emerged. RRT-connect [19] simultaneously expands two trees from the initial and target states and then connects the two at the appropriate position, speeding up the pathfinding process. Kang et al. proposed a triangular inequality-based rewiring method for the RRT-connect algorithm [20]. The improved algorithm shows a shorter path length than RRT-connect. The above algorithms have probabilistic completeness, i.e., as the number of iterations approaches infinity, the probability of finding a feasible solution tends towards one. However, none of these methods can ensure optimality. RRT* [21] solves the problem by adopting the ChooseParent and Rewire procedures, which provide asymptotic optimality. An algorithm with asymptotic optimality means that as the number of iterations approaches infinity, the algorithm guarantees that the probability of finding an optimal solution approaches one [22].

Although RRT* can improve the initial solution to the optimum, it has a slow convergence rate due to the large amount of computation caused by the continuous increase in the number of vertices. Therefore, enhancing the convergence rate of RRT* is of great significance and has attracted extensive attention. Jonathan et al. proposed Informed-RRT* [23] based on direct sampling. This method defines the acceptable sampling space as a hyper-ellipsoid and samples directly from it. By narrowing the sampling region, Informed-RRT* may converge to the optimal path rapidly. However, when the relevant hyper-ellipsoid exceeds the region of the motion planning problem, the algorithm will not be applicable. Quick-RRT* [24] uses triangular inequality to improve the ChooseParent and Rewire procedures and converges faster than RRT*. The downside is that it wastes many computing resources on useless vertices, which do not help in finding the optimal path. MOD-RRT* [25] introduces a re-planning procedure to improve the performance of the algorithm, which is used to modify unfeasible paths to generate high-quality paths. GMR-RRT* [26] uses Gaussian mixture regression (GRM). This algorithm learns the human driving path and finds key features through GRM to form a probability distribution to guide sampling. Jun et al. proposed Feedback-RRT* (F-RRT*) [27], with a data-driven risk network and feedback module. F-RRT* can use the information extracted from situation data to constrain the growth of the random tree and make biased adjustments to improve planning efficiency.

This paper proposes Metropolis-RRT* (M-RRT*) for the motion planning of mobile robots. Based on the principle of the Metropolis acceptance criterion, on one hand, in the initial path estimation phase of the algorithm, an asymptotic vertex acceptance criterion is developed, which preferentially accepts vertices close to the target state. On the other hand, in the optimal path search phase, a nonlinear dynamic vertex acceptance criterion is also developed, which preferentially accepts vertices that may lower the current optimal path cost. Therefore, M-RRT* substantially reduces the number of vertices required for planning, thereby obtaining the initial path more efficiently and converging to the optimal path faster.

The M-RRT* algorithm proposed in this study is applied to solve the motion planning problem of mobile robots, with the following main contributions:

- Propose an asymptotic vertex acceptance criterion in the initial path estimation phase of the algorithm to effectively reduce the time of finding the initial path and make the algorithm start searching for the optimal path earlier.
- Propose a nonlinear dynamic vertex acceptance criterion in the optimal path search phase of the algorithm. This criterion can reduce the number of vertices in the algorithm that are not capable of improving the current path so as to rapidly converge to the optimal path.
- The experimental results in three common types of environments show that the proposed algorithm has an outstanding performance. It takes less time to find the initial path and has a fast convergence rate in the cluttered environment and the regular environment. Due to the complexity of the maze, M-RRT* does not improve much in the initial path estimation phase, but more importantly, its convergence speed still increases significantly.

The rest of this paper is structured as follows. Section 2 introduces the definition of the motion planning problem and related algorithms. Section 3 explains the proposed algorithm in detail. Section 4 analyzes the simulation results compared with RRT*, Informed-RRT*, and Q-RRT*. Section 5 summarizes this paper and looks forward to future research plans.

## 2. Problem Definition and Related Work

This section defines the motion planning problem and details the basic algorithm of M-RRT*. To understand the comparative analysis in Section 4, this section also provides an explanation of Informed-RRT* and Q-RRT*.

### 2.1. Problem Definition

When a robot performs a given task, the robot itself or a certain part moves from the initial state to the target state. Motion planning can provide the robot with a collision-free path from the initial state to the target state, which is called a feasible path. As mentioned in the previous section, motion planning has been applied to various robots, and the planning results directly affect the efficiency of the robot in completing a given task. Planners require motion planning not only to be able to search for an optimal path but also to make the planning process as fast as possible. The mathematical definition of motion planning is described in detail below.

Let X ⊆ ℝ*^d^* be a *d*-dimensional configuration space, X*_obs_* ⊂ X be the obstacle region, and X*_free_* = *cl*(X\X*_obs_*) be the obstacle-free region, where *d* ∈ ℕ, *d* ≥ 2, and *cl*(·) refers to the closure of the set. *x_init_* ∈ X*_free_* is the initial state, and X*_goal_* ⊂ X*_free_* is the goal region, where the motion planning will be completed if the object reaches this region. The continuous function *σ*: [0, 1] ↦ X is called a path. A path *σ*: [0, 1] ↦ X*_free_* is collision-free if ∀*τ* ∈ [0, 1], *σ*(*τ*) ∈ X*_free_*.

**Definition** **1.** *(Feasible motion planning) Given a motion planning problem {x_init_, X_goal_, X_obs_}, find a collision-free path σ, where σ(0) = x_init_, σ(1) ∈ X_goal_. This path is called a feasible path.*

**Definition** **2.** (Optimal motion planning) Given a motion planning problem {x_init_, X_goal_, X_obs_}, find a feasible path σ that minimizes the cost c(σ*), i.e., c(σ*) = min{c(σ*): σ ∈ Σ}, where c(σ) is the cost of a feasible path σ in X measured by the Euclidean distance, and Σ is the set of all feasible paths.

**Definition** **3.** (Fast motion planning) Given a motion planning problem {x_init_, X_goal_, X_obs_}, find the optimal feasible path σ* in the shortest possible time.

### 2.2. RRT*

RRT* is a sampling-based motion planning algorithm. Since RRT* is an extended algorithm of RRT, a brief description of its basic algorithm is required first. RRT gradually explores the collision-free path from the initial state *x_init_* which is the only vertex with no edges. In each iteration, *x_rand_* is randomly sampled in the collision-free space X*_free_*. Then, we find the vertex *x_nearest_* closest to the sample *x_rand_* from the tree based on the Euclidean metric. After finding *x_nearest_*, extend a distance from *x_nearest_* towards *x_rand_* to obtain a new vertex *x_new_*, and this distance *η* is called the step size. Determine whether the path from *x_nearest_* to *x_new_* collides with obstacles based on the environmental information. If a collision occurs, the path is discarded, and the next iteration proceeds. If no collision occurs, it is a feasible path, and this path and the vertex *x_new_* are added to the tree. Repeat the above steps until a feasible path is found and output. As shown in Algorithm 1, the major difference from RRT is that RRT* contains the ChooseParent and Rewire procedures, which makes it asymptotically optimal.

**Figure 1:**
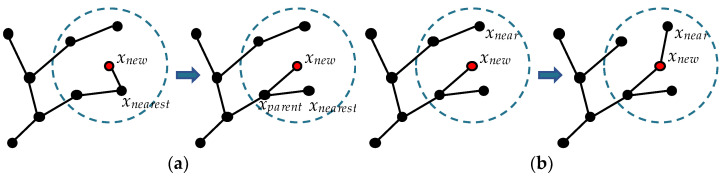
(**a**) ChooseParent (RRT*); (**b**) Rewire (RRT*). (red dot: *x_new_*, dashed blue circle: *Near*(*x_new_*.)).

**Algorithm 1** RRT*1:*G* ← (*V*, *E*); *V* ← *x_init_*; 2:**for** *i* = 1 to *n* **do**3: *x_rand_* ← *Sample*(*i*);4: *x_nearest_* ← *Nearest*(*V*, *x_rand_*);5: (*x_new_*, *σ*) ← *Steer*(*x_nearest_*, *x_rand_*);6: **if** *CollisionFree*(*σ*) **then**7:  *X_near_* ← *Near*(*V*, *x_new_*);8:  (*x_parent_*, *σ_parent_*) ← *ChooseParent*(*X_near_*, *x_nearest_*, *x_new_*, *σ*);9:  *V* ← *AddVertex*(*x_new_*);10:  *E* ← *AddEdge*(*x_parent_*, *x_new_*);11:  *G* ← *Rewire*(*G*, *x_new_*, *X_near_*); 12: **end if**13:
**end for**
14:**return** *G*;

In the ChooseParent procedure, RRT* searches for a vertex in a hypersphere of a specific radius centered at *x_new_* such that the path through this vertex to *x_new_* has the lowest cost. It is then used as the parent vertex of *x_new_*, as shown in Figure 1a. In the Rewire procedure, the vertices in the hypersphere are represented by *x_near_* in turn. Compare the cost of the current path to *x_near_* with the cost of the path through *x_new_* to *x_near_* until every vertex in the hypersphere has been compared. During each comparison, if the path through *x_new_* is less costly, change its wiring, as shown in Figure 1b. Algorithm 2 shows the ChooseParent procedure, and Algorithm 3 shows the Rewire procedure.

**Algorithm 2** ChooseParent(*X_near_*, *x_nearest_*, *x_new_*, *σ_nearest_*)1:*x_min_* ← *x_nearest_*;2:*σ_min_* ← *σ_nearest_*;3:*c_min_* ← *Cost*(*x_min_*) + *Cost*(*σ_min_*);4:**for** each *x_near_* ∈ *X_near_* **do**5: *σ* ← *Connect*(*x_near_*, *x_new_*);6: *c* ← *Cost*(*x_near_*) + *Cost*(*σ*);7: **if** *c* < *c_min_* **then**8:  **if**
*CollisionFree*(*σ*) **then**9:   *x_min_* ← *x_near_*;10:   *σ_min_* ← *σ*;11:   *c_min_* ← *c*;12:  **end if**13: **end if**14:
**end for**
15:**return** (*x_min_*, *σ_min_*);

**Algorithm 3** Rewire(*G*, *x_new_*, *X_near_*)1:**for** each *x_near_* ∈ *X_near_* **do**2: *σ* ← *Connect*(*x_new_*, *x_near_*);3: **if** *Cost*(*x_new_*) + *Cost*(*σ*) < *Cost*(*x_near_*) **then**4:  **if**
*CollisionFree*(*σ*) **then**5:   *G* ← *Reconnect*(*G*, *x_new_*, *X_near_*, *σ*);6:  **end if**7: **end if**8:
**end for**
9:**return***G*;

The following briefly describes the basic procedures used in the RRT* algorithm.

- *Sample*: Returns a random sample from X.
- *Nearest*: Given a graph *G* = (*V*, *E*) and a state *x*, it returns the vertex closest to *x* according to the given Euclidean distance function.
- *Steer*: Given two states *x_s_*, *x_t_* ∈ X, it extends *x_s_* to *x_t_* by a given distance to obtain the state *x_d_* ∈ X. Then, it returns *x_d_* and the path from *x_s_* to *x_t_*.
- *CollisionFree*: Checks whether the given path *σ* is a feasible path.
- *Near*: Given a graph *G* = (*V*, *E*) and a state *x*, it sets a hypersphere of a given radius centered at *x* and returns the set *X_near_* of vertices in *V* that are contained in this hypersphere.
- *AddVertex*: Given a state *x*, it adds *x* to the graph.
- *AddEdge*: Given two states *x_s_*, *x_t_* ∈ X, it adds the path from *x_s_* to *x_t_* to the graph.
- *Connect*: Given two states *x_s_*, *x_t_* ∈ X, it returns the path from *x_s_* to *x_t_*.
- *Reconnect*: Given a graph *G* = (*V*, *E*), two states *x_s_*, *x_t_* ∈ X, and a path *σ* from *x_s_* to *x_t_*, it replaces the parent vertex of *x_t_* with *x_s_* and adds *σ* to graph *G*.
- RRT searches for a feasible path by imitating a randomly grown tree, but this algorithm cannot find the optimal path. Unlike RRT, RRT* has asymptotic optimality by introducing the ChooseParent and Rewire procedures. However, due to the frequent running of these two procedures, the convergence speed of RRT* is slow.

### 2.3. Informed-RRT*

**Figure 2:**
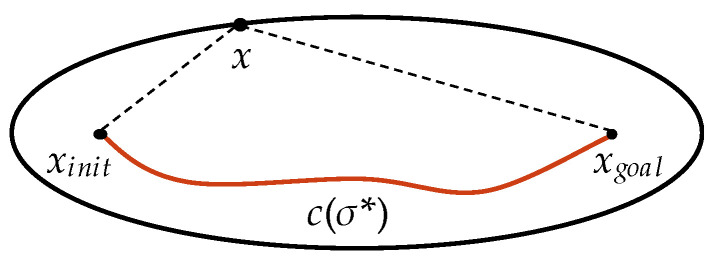
Hyper-ellipsoid of Informed-RRT*.

Informed-RRT* has the same procedure as RRT* until the initial path is found. In the optimal path search phase, Informed-RRT* constructs a hyper-ellipsoid with focal points *x_init_* and *x_goal_*. It then samples directly inside the hyper-ellipsoid to find the optimal path. The sampling region of the set of vertices satisfies

(1) $$\|x-x_{init}\|+\|x_{goal}-x\|\leq c(\sigma *),$$

where *x* is the random sample, σ* is the best path with the minimum cost at the current time, c(σ*) is the cost of σ*, and ‖x−y‖ is the Euclidean distance between *x* and *y*. Figure 2 shows the hyper-ellipsoid set by Informed-RRT*. When the current best solution is updated, its cost c(σ*) is also updated. Therefore, the sampling region, i.e., the hyper-ellipsoid, gradually becomes smaller, and the probability of the sample being on the optimal path is greater.

The direct sampling method significantly increases the convergence rate of In-formed-RRT*. However, Informed-RRT* does not improve the efficiency of finding the initial solution. Moreover, although Informed-RRT* converges faster than RRT*, its efficiency decreases when the region of the hyper-ellipsoid exceeds the configuration space.

### 2.4. Q-RRT*

**Figure 3:**
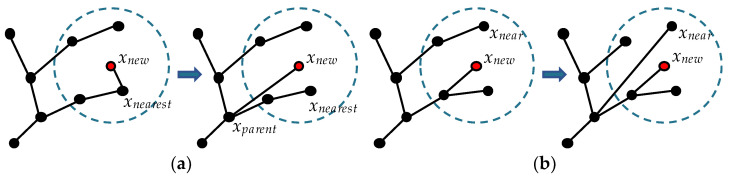
(**a**) ChooseParent (Q-RRT*); (**b**) Rewire (Q-RRT*).

Quick-RRT* (Q-RRT*) makes adjustments to the ChooseParent and Rewire procedures. In the ChooseParent procedure, RRT* searches for the potential parent vertex of *x_new_* from *X_near_*, while the search scope of Quick-RRT* also includes the ancestors of *X_near_*. Quick-RRT* defines a depth in advance, which is used to set the number of vertices backtracked when searching for ancestors. In Figure 3a, through the ChooseParent procedure, *x_new_* selects *x_parent_* as the parent vertex, and *x_parent_* is the ancestor vertex of *x_nearest_*, with a depth of 2. Similar to the ChooseParent procedure, the Rewire procedure also considers the ancestor of the vertex *x_new_*, as shown in Figure 3b.

Q-RRT* essentially improves the structure of the random tree. Compared with RRT*, the ChooseParent and Rewire procedures of Q-RRT* make the path more optimized. Although this algorithm can improve the structure, it needs to search more vertices. If most of the vertices do not meet the connection conditions, it means that the algorithm wastes a lot of time.

## 3. Methods

This section describes the proposed M-RRT* algorithm in detail. Most algorithms based on RRT* are divided into two phases: finding the initial path and finding the optimal path. The initial path is the first feasible path found by the algorithm. Since the initial path of the sampling-based algorithm is not optimal, it is necessary to continue sampling to find the optimal path.

Similarly, M-RRT* includes the initial path estimation and optimal path search phases. Inspired by the Metropolis acceptance criterion, we design different vertex acceptance criteria in the above two phases to calculate the retention probability of the new vertex generated by each iteration. M-RRT* uses the asymptotic vertex acceptance criterion in the initial path estimation phase. These enable the algorithm to obtain the initial solution faster. In the optimal path search phase, M-RRT* uses the nonlinear dynamic vertex acceptance criterion to improve the efficiency of finding the optimal solution. The following briefly introduces the Metropolis acceptance criterion.

### 3.1. Metropolis Acceptance Criterion

The core of the Metropolis acceptance criterion is the limited acceptance of inferior solutions. It is generally used in the simulated annealing algorithm to calculate the acceptance probability of a solution. The probability of a new solution being accepted is given by

(2) $$P=\left\{\begin{array}{c} \;\;\;1\;\;,\Delta E<0\\ \exp(-\Delta E/T),\Delta E\geq 0 \end{array}\right.$$

where exp(·) refers to an exponential function, *T* is temperature, defined as the control parameter, *E* is internal energy, defined as the objective function, and ΔE=E(n+1)−E(n). The energy of the current state *n* of the system is *E*(*n*), and the energy of the next state is *E*(*n* + 1). The algorithm tries to gradually decrease the value of the objective function as the temperature *T* decreases until *E* tends to the global minimum, just like the solid annealing process. A new solution will change the internal energy at the corresponding temperature, and the size of the change is ΔE. It can be seen from (2) that if ΔE<0, the new solution is accepted. If ΔE≥0, the new solution is accepted by probability P=exp(−ΔE/T). The following describes the effect of the variation of *T* on the candidate solutions.

- If *T* is a fixed value, the probability of accepting the candidate solution that reduces the value of *E* is greater than the probability of accepting the solution that increases the value.
- If *T* gradually decreases, the probability of accepting the candidate solution that increases the value of *E* also decreases.
- If *T* tends to zero, the algorithm only accepts the candidate solution that reduces the value of *E*.

Generally, a large *T* will perform a global search, but the computational cost will increase. While a small *T* will search locally and make a fast convergence rate, it tends to trap the algorithm in a local optimum.

### 3.2. Asymptotic Vertex Acceptance Criterion

The ChooseParent and Rewire procedures make RRT* asymptotically optimal, but frequent collision detection and searching for neighboring vertices increase the algorithm’s complexity. Therefore, this paper introduces the asymptotic vertex acceptance criterion into the initial path estimation phase. After M-RRT* samples a new vertex, the impact of this vertex on finding the initial solution is quantified as a prediction value through a prediction function, and the probability of retaining this vertex is calculated according to the predicted value. If the new vertex is not retained, M-RRT* does not perform the subsequent calculation steps of this iteration and directly starts the next iteration. This method enables the random tree to grow to the target region faster; thus, an initial feasible solution is found more quickly.

**Figure 4:**
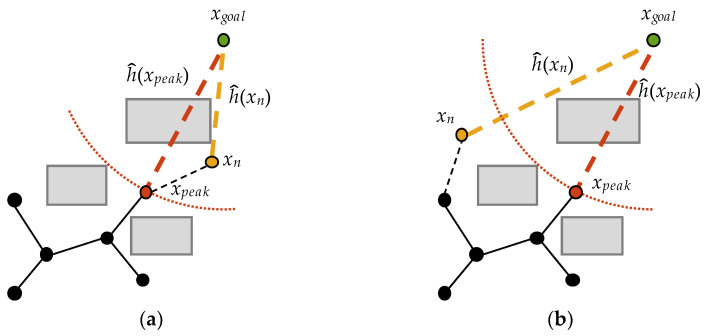
Tree construction of case (**a**) and case (**b**).

To facilitate the description, we denote the target state by *x_goal_*, the new vertex generated by the nth iteration by *x_n_*, and the vertex closest to the *x_goal_*, i.e., the vertex with the smallest Euclidean distance to *x_goal_*, by *x_peak_*. We can describe the process of finding an initial path as making *x_peak_* asymptotically approach *x_goal_* until it reaches the location of *x_goal_*. The admissible heuristic estimate $\hat{h}$ is cost-to-go, i.e., the cost to go from any state to the goal. The prediction function for *x_n_* is as follows,

(3) $$C(n)=\hat{h}(x_{n})-\hat{h}(x_{peak})$$

where $\hat{h}(x_{n})$ is the Euclidean distance from *x_n_* to *x_goal_* and $\hat{h}(x_{peak})$ is the Euclidean distance from *x_peak_* to *x_goal_*. Similar to the Metropolis acceptance criterion, we can judge whether *x_n_* is closer to *x_goal_* by *C*(*n*). As shown in Figure 4, one of the following two cases occurs during the *n*th iteration.

- If C(n)<0, *x_n_* lies in a spherical range with *x_goal_* as the center and $\hat{h}$(*x_peak_*) as the radius; this means that *x_n_* is closer to *x_goal_* than *x_peak_*. Therefore, *x_n_* is likely to play a positive role in finding the initial feasible path, at which point *x_n_* is retained with a probability of one and is defined as *x_peak_*, as shown in Figure 4a.
- If C(n)≥0, *x_n_* is outside the spherical range, and it is further away from *x_goal_* than *x_peak_*. In this case, the probability of retaining *x_n_* must be calculated based on the predicted value, as shown in Figure 4b.

It is worth noting that at the beginning of M-RRT*, the only vertex in the state space, *x_init_*, which is the start state, is at the same position as *x_peak_*. To quickly estimate the initial feasible solution, the heuristic value of each extended vertex preferably shows a downward trend. However, when C(n)≥0, *x_n_* must be accepted with a certain probability to prevent the algorithm from falling into a local optimum. Therefore, this paper proposes the asymptotic vertex acceptance criterion combined with the prediction function. The probability of accepting *x_n_* is

(4) $$P_{n}=\left\{\begin{array}{c} \;\;\;1\;\;\;\;,C(n)<0\\ \exp(-C(n)/\hat{h}(x_{init})),C(n)\geq 0 \end{array}\right.$$

where $\hat{h}(x_{init})$ is the Euclidean distance from *x_init_* to *x_goal_*. Compared with *x_peak_*, it is clear that the probability of acceptance is smaller if *x_n_* is further away from the goal, and vice versa. Since $\hat{h}$(*x_init_*) is the cost of the theoretical optimal path, it has a reference value for the calculation of the acceptance probability of vertices. In this paper, we define $\hat{h}$(*x_init_*) as the control parameter.

In this phase, the probability of accepting each expanded vertex is greater than zero; thus, M-RRT* is capable of jumping out of the local optimum, but in some complex environments, it may take longer. We design a method to jump out of the local optimum quickly. If the value of $\hat{h}$(*x_peak_*) does not change after using Equation (4) to calculate the probability 20 times, the algorithm is considered to fall into the local optimum. The acceptance probability of the subsequent extended new vertex is one until the value of $\hat{h}$(*x_peak_*) changes. Then, the random tree is closer to the goal, indicating that the algorithm jumps out of the local optimum and continues to use Equation (4).

The asymptotic vertex acceptance criterion makes M-RRT* obtain the initial feasible path faster. At the same time, the algorithm performs the optimal path search phase earlier and improves the overall efficiency.

### 3.3. Nonlinear Dynamic Vertex Acceptance Criterion

Given an optimal path *σ* from *x_init_* through *x* ∈ X; to *x_goal_*, *g*(*x*) is equal to the cost of the optimal path from *x_init_* to *x*, *h*(*x*) is equal to the cost of the optimal path from *x* to *x_goal_*, and the cost of *σ* is c(σ)=g(x)+h(x). $\hat{g}(x)$ and $\hat{h}(x)$ are admissibility heuristics for g(x) and h(x), respectively. In the problem of solving the optimal path length in ℝ*^d^*, the Euclidean distance applies to both heuristics.

An algorithm based on RRT* will continue to iterate to search for the optimal path after completing the process of finding the initial feasible path. When *x_n_* is generated at the nth iteration, there exists a feasible path σ* with the minimum cost at the current time. To make σ* approach the optimal path asymptotically, M-RRT* not only estimates the path cost from the new vertex *x_n_* to *x_goal_* but also considers the path cost from *x_init_* to *x_n_*. Next, we will introduce the nonlinear dynamic vertex acceptance criterion in detail, which is applied to the optimal path search phase of M-RRT*.

**Figure 5:**
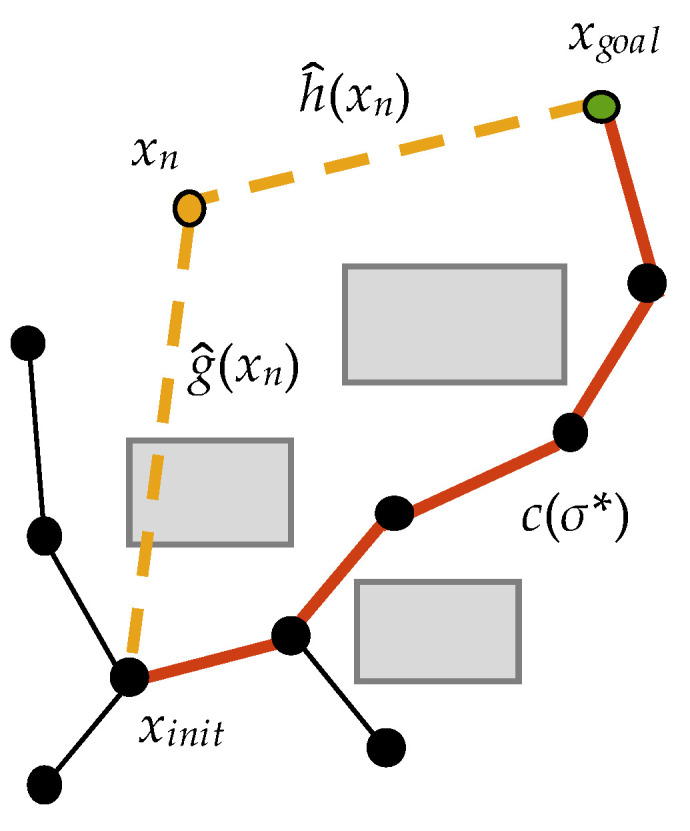
Tree construction.

In Figure 5, $\hat{g}(x_{n})$ is the Euclidean distance from *x_init_* to *x_n_*, $\hat{h}(x_{n})$ is the Euclidean distance from *x_n_* to *x_goal_*, σ* is the best path with the minimum cost at the current time, and c(σ*) is the cost of σ*. Obviously, $\hat{g}(x_{n})+\hat{h}(x_{n})$ is the cost of the theoretically optimal path through *x_n_*. 

If $c(\sigma *)<\hat{g}(x_{n})+\hat{h}(x_{n})$, it indicates that the cost of any path through *x_n_* cannot be less than c(σ*). Then, the probability of accepting *x_n_* is $P_{n}=0$, and M-RRT* starts the next iteration directly. Therefore, it removes vertices that are entirely impossible to improve the path length, avoiding wasting time on these useless vertices. 

**Figure 6:**
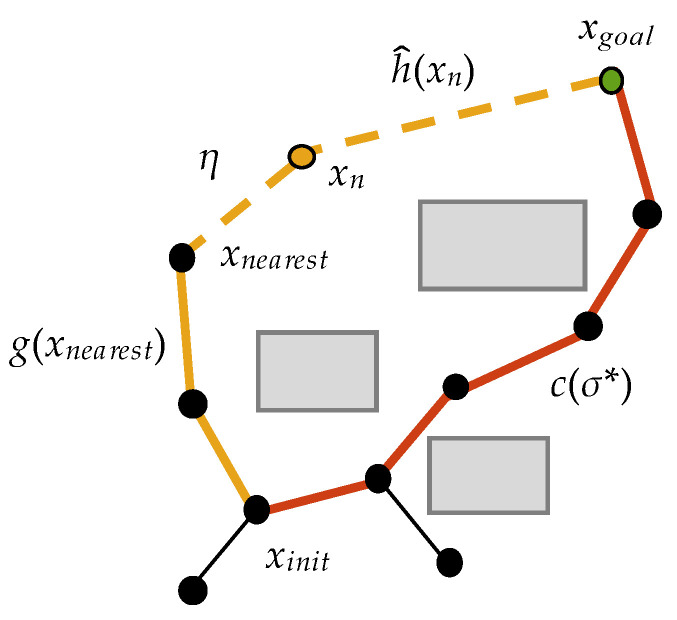
Tree construction. (*g*(*x_n_*) = *g*(*x_nearest_*) + *η*.).

If $c(\sigma *)\geq \hat{g}(x_{n})+\hat{h}(x_{n})$, we must refer to the actual cost *g*(*x_n_*) from *x_init_* to *x_n_* and establish the prediction function

(5) $$C(n)=g(x_{n})+\hat{h}(x_{n})-c(\sigma *)$$

where *g*(*x_n_*) is the actual cost of the path from *x_init_* to *x_n_*, and $\hat{h}(x_{n})$ is the estimated cost from *x_n_* to *x_goal_*, which is the Euclidean distance. Similar to the Metropolis acceptance criterion, we predict whether the new vertex *x_n_* can improve the current optimal path σ* by *C*(*n*). As shown in Figure 6, *x_n_* is generated by extending the distance *η* from the nearest vertex *x_nearest_* on the random tree; thus, the actual cost $g(x_{n})=g(x_{nearest})+\eta .$ As mentioned in the previous section, $\hat{h}(x_{n})$ is the estimated cost of *x_n_* to *x_goal_*. One of the following two cases occurs during the nth iteration.

- If C(n)<0, the predicted value is lower than the current minimum cost c(σ*). Therefore, *x_n_* is likely to play a positive role in finding the optimal path, and the probability of retaining *x_n_* is one.
- If C(n)≥0, the probability of retaining *x_n_* must be calculated based on the predicted value.

In the case of C(n)≥0, the probability of retaining *x_n_* cannot be zero, because after the ChooseParent and Rewire procedures, the actual cost from *x_init_* to *x_n_* may be reduced. In this case, *x_n_* has the possibility of optimizing the path, but, of course, the larger the value of the prediction function, the smaller the possibility. Combined with the prediction function, the probability of accepting a vertex is

(6) $$P_{n}=\left\{\begin{array}{c} \;\;\;1\;\;\;\;\;,C(n)<0\\ \exp(-\frac{C(n)}{c(\sigma *)/\ln(n-N-1+e)}),C(n)\geq 0 \end{array}\right.$$

where c(σ*) is the current minimum cost, *n* is the current number of iterations, and *N* is the number of iterations when σ* is found. Therefore, *N* changes dynamically with the update of σ*, and the next iteration number *n* after the update is equal to *N* + 1. The nonlinear dynamic cost {c(σ*)/ln(n−N−1+e)} increases from c(σ*), and then the algorithm gradually reduces the probability of accepting vertices that make C(n)≥0. The nonlinear dynamic vertex acceptance criterion makes the algorithm sample in an extensive range in the early stage to prevent falling into the local optimum. As the number of iterations increases, the retained samples are more targeted, enabling the algorithm to quickly converge and find the optimal path. Moreover, there is always a probability of global sampling, allowing M-RRT* to explore a wider area while converging quickly, ensuring its asymptotic optimality.

### 3.4. M-RRT*

Compared with RRT*, M-RRT* adopts the asymptotic vertex acceptance criterion and nonlinear dynamic vertex acceptance criterion in finding the initial path and optimal path, respectively. It not only preserves the probability of global sampling but also selectively accepts vertices conducive to finding solutions and removes many useless vertices. M-RRT* prunes the random tree, significantly improving computational efficiency and reducing memory usage. Algorithm 4 shows the pseudocode of M-RRT*.

**Algorithm 4** M-RRT*1:*G* ← (*V*, *E*); *V* ← *x_init_*; *x_peak_* ← *x_init_*; *N* ← 0;2:$\hat{h}$(*x_peak_*) ← *Dis*(*x_peak_*, *x_goal_*);3:$\hat{h}$(*x_init_*) ← *Dis*(*x_init_*, *x_goal_*);4:**for** *i* = 1 to *n* **do**5: *x_rand_* ← *Sample*(*i*);6: *x_nearest_* ← *Nearest*(*V*, *x_rand_*);7: (*x_n_*, *σ*) ← *Steer*(*x_nearest_*, *x_rand_*);8: **if** *CollisionFree*(*σ*) **then**9:  **if** *N* = 0 **then**10:   *AVAC*(*x_n_*, $\hat{h}$(*x_peak_*), $\hat{h}$(*x_init_*));11:  **else**12:   *NDVAC*(*x_n_*, *x_nearest_*, *n*, *N*, σ*);13:  *X_near_* ← *Near*(*V*, *x_n_*);14:  (*x_parent_*, *σ_parent_*) ← *ChooseParent*(*X_near_*, *x_nearest_*, *x_n_*, *σ*);15:  *V* ← *AddVertex*(*x_n_*);16:  *E* ← *AddEdge*(*x_parent_*, *x_n_*);17:  *G* ← *Rewire*(*G*, *x_n_*, *X_near_*); 18:  (σ*, *N*) ← *NearGoal*(*G*, *x_n_*, *n*);19: **end if**20:
**end for**
21:**return** *G*;

The following briefly describes the basic procedures used in the M-RRT* algorithm.

**Figure 7:**
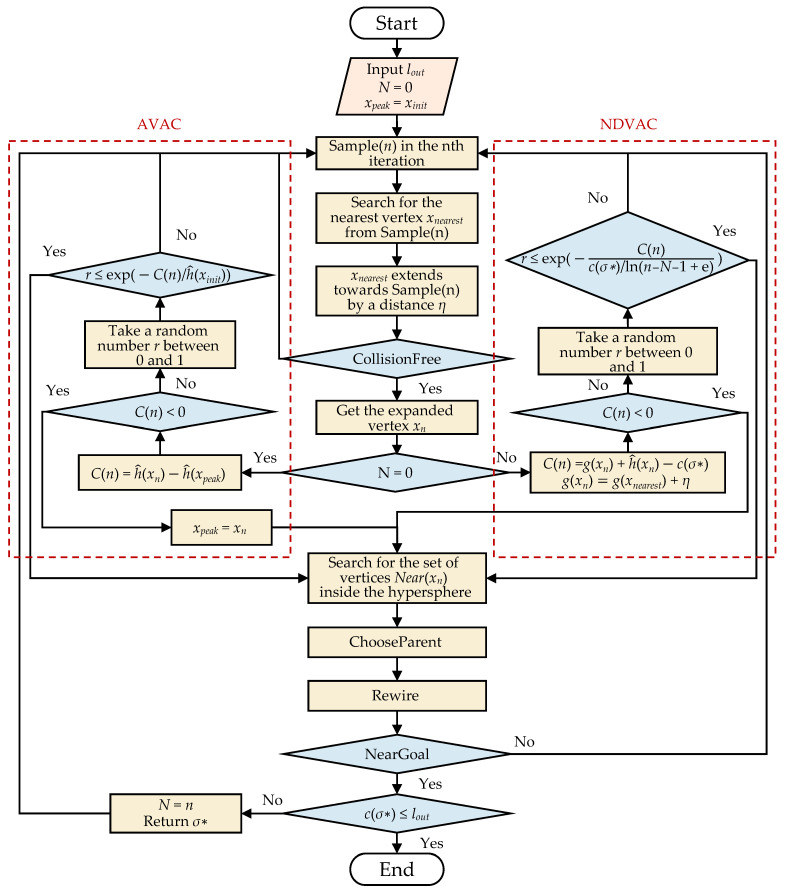
Flow chart of M-RRT*.

- *Dis*: Given two states *x_s_*, *x_t_* ∈ X, it returns the Euclidean distance between *x_s_* and *x_t_*.
- *AVAC*: Asymptotic vertex acceptance criterion.
- *NDVAC*: Nonlinear dynamic vertex acceptance criterion.
- *NearGoal*: Given a graph *G* = (*V*, *E*), a state *x*, and the current number of iterations, if *x* is within a given range around *x_goal_*, this procedure returns the feasible path σ* with the least cost and the current number of iterations *N*.
- Figure 7 shows the flow chart of M-RRT*, and *l_out_* is the set path length to which to converge. The ChooseParent and Rewire procedures have been described in related work, so they are not shown in detail in the figure. The NearGoal procedure has also been introduced in this section.

## 4. Simulations

Since RRT* is the basic algorithm of the algorithm proposed in this paper, Informed-RRT* is widely recognized as an efficient RRT-based algorithm, and Q-RRT* is a newly proposed correlation algorithm, this paper uses them for comparative analysis. Q-RRT* has the best efficiency when the depth is 2, so 2 is chosen as the depth in this paper.

### 4.1. Environment Configuration and Indicator Description

**Figure 8:**
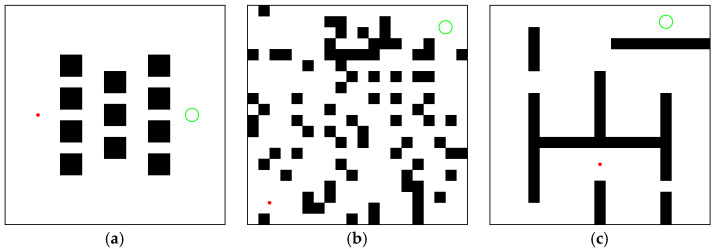
Environments for the simulations. (**a**) Regular environment; (**b**) Cluttered environment; (**c**) Maze. (Red dot: start state, green circle: goal region.).

M-RRT* is compared with the above three algorithms in three environments of the same size of 100 × 100. The simulation environments are shown in Figure 8. Each algorithm was run 100 times because of the randomness of the sampling-based algorithms. The system and resource characteristics used in the simulation implementation are shown in Table 1.

In this section, all algorithms were simulated with the same parameters. Two evaluation indicators are used to compare the performances of the algorithms. Firstly, some problems require finding a feasible solution for motion planning in a short time; hence, this paper compares the time of the four algorithms to find the initial path. We define this time as the index ‘*t_init_*’. Secondly, four algorithms have asymptotic optimality; thus, they can all find the optimal path as the number of iterations approaches infinity. Due to the randomness of the sampling-based algorithm, this paper first determines the optimal path length in each environment and then compares the time it takes for the algorithms to converge to an approximate optimal path. We define this time as the index ‘*T*_5%_’. The length of the approximate optimal solution is ‘1.05∗*l_optimal_*’, where ‘*l_optimal_*’ is the length of the optimal solution.

Again, because of randomness, the statistical results of 100 runs of each algorithm are described by boxplots, and the specific values are shown in tables. In a boxplot, a line in the middle of the box represents the median of the data. The bottom and top of the box are the upper and lower quartiles of the data, respectively. Therefore, the height of the box reflects the fluctuation of the data to some extent. The median can show the performance of the randomness algorithms. In addition, the paper also describes the mean, maximum, and minimum to prove the efficiency and stability of the algorithms. The difference between the upper and lower quartiles is called the interquartile range (IQR). Values that are 1.5 times IQR larger than the upper quartile, or 1.5 times IQR less than the lower quartile, are classified as outliers and shown as rhombus in the boxplot.

### 4.2. Regular Environment

**Figure 9:**
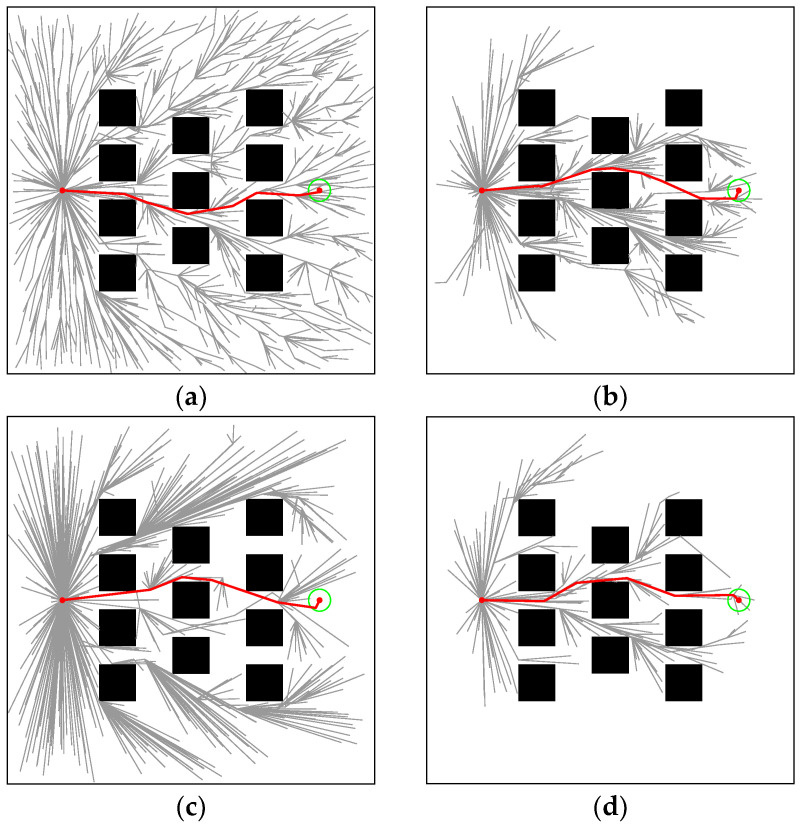
Performance of the four algorithms in the regular environment. (**a**) RRT*. (**b**) Informed-RRT*. (**c**) Q-RRT*. (**d**) M-RRT*. (Red line: approximate optimal path, green circle: goal region.).

The regular environment is shown in Figure 8a. In Figure 9, the paths generated by RRT* (*t_init_* = 0.1093, *T*_5%_ = 1.093), Informed-RRT* (*t_init_* = 0.0781, *T*_5%_ = 0.453), Q-RRT* (*t_init_* = 0.6876, *T*_5%_ = 0.7059), and M-RRT* (*t_init_* = 0.0625, *T*_5%_ = 0.1406) are shown. In the regular environment, *l_optimal_* = 70.9268. The grey lines in each figure are the paths explored by the algorithm through the extended vertices, and the red line is the approximate optimal path. RRT* randomly sampled the entire state space; hence, its vertices grew in any direction. Unlike RRT*, once the initial path was found, Informed-RRT* sampled only inside the hyper-ellipsoid, with the start and target states as the foci. Therefore, most of the vertices of Informed-RRT* appear to be inside an ellipse. Q-RRT* optimizes the structure of the random tree so that the path between its vertices and the initial vertex is as straight as possible. Since M-RRT* preferentially accepted vertices that are conducive to searching for solutions and had a limited acceptance of vertices that provide inferior solutions, it generated fewer vertices than the other two algorithms. 

**Figure 10:**
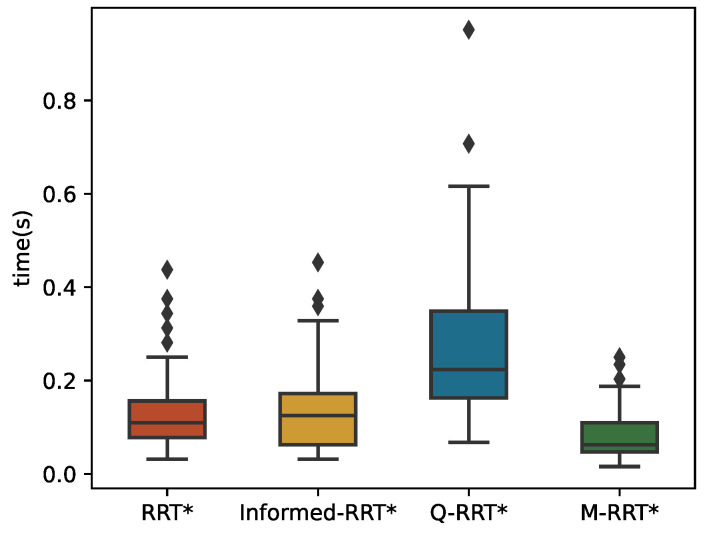
*t_init_* in the regular environment.

**Figure 11:**
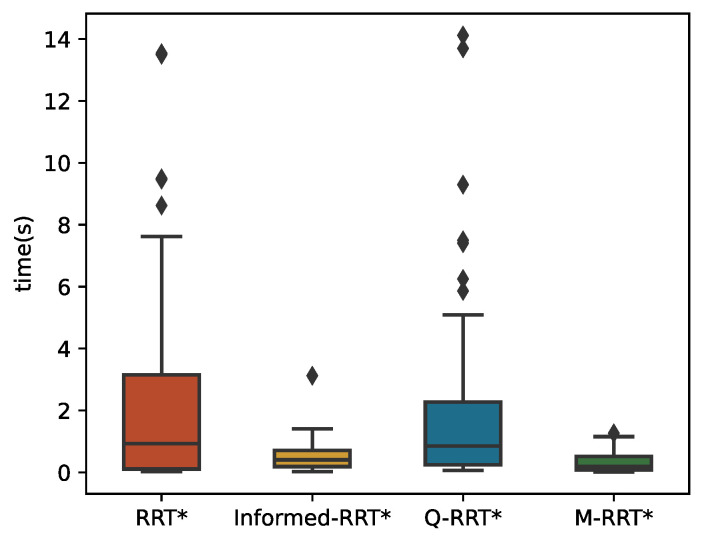
*T*_5%_ in the regular environment.

The 100 simulation results of *t_init_* and *T*_5%_ are described by boxplots, as shown in Figure 10 and Figure 11, respectively. Table 2 counts the specific numerical values of the simulation results. For the convenience of the comparison, the data in the table are generally displayed to the fourth decimal place. If the size of the same indicator for the algorithms is very close, it shows the decimal place where their numbers are different. The two minimum values of the three algorithms are the same because the length of the initial path is less than 1.05∗*l_optimal_*. Due to the regular distribution of obstacles in this environment and the small distance between the initial and goal states, the above situation is common for sampling-based algorithms. Although RRT* and Informed-RRT* have identical procedures for finding initial solutions, they are less stable, as shown in Figure 10. Since Q-RRT* consumes more computing resources on the optimized path, it takes a long time to find the initial path. In addition, M-RRT* takes less time to find the initial solution. It is clear from Figure 11 that M-RRT* converges faster than the other three algorithms. Based on the above analysis, M-RRT* outperforms RRT*, Informed-RRT*, and Q-RRT* in the regular environment.

### 4.3. Cluttered Environment

**Figure 12:**
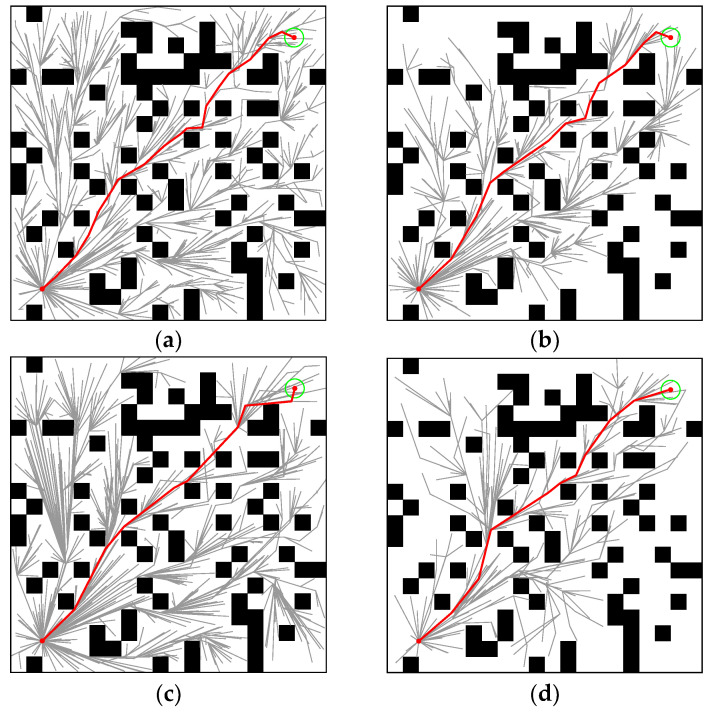
Performance of the four algorithms in the cluttered environment. (**a**) RRT*. (**b**) Informed-RRT*. (**c**) Q-RRT*. (**d**) M-RRT*. (Red line: approximate optimal path, green circle: goal region.).

The cluttered environment is shown in Figure 8b. In Figure 12, the paths generated by RRT* (*t_init_* = 0.337, *T*_5%_ = 3.1296), Informed-RRT* (*t_init_* = 0.346, *T*_5%_ = 1.6216), Q-RRT* (*t_init_* = 1.0265, *T*_5%_ = 4.6131), and M-RRT* (*t_init_* = 0.2852, *T*_5%_ = 0.8666) are shown. In the cluttered environment, *l_optimal_* = 114.8326. RRT* required many vertices to explore the state space fully, while M-RRT* generated the fewest vertices to converge to the approximate optimal solution.

**Figure 13:**
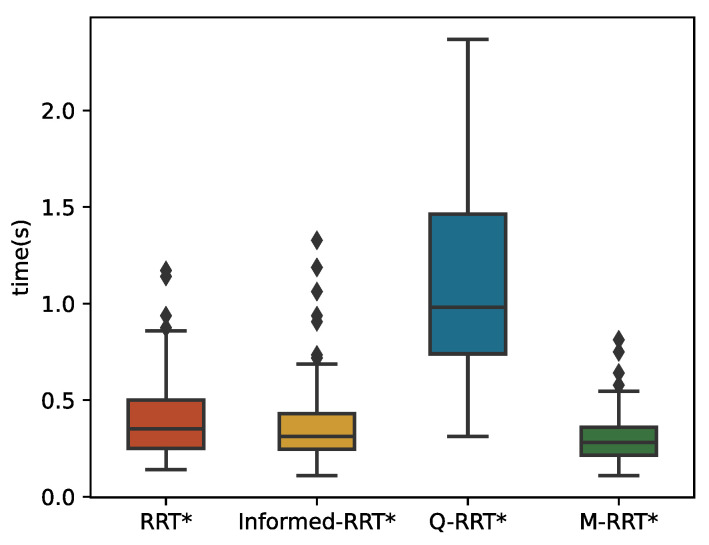
*t_init_* in the cluttered environment.

**Figure 14:**
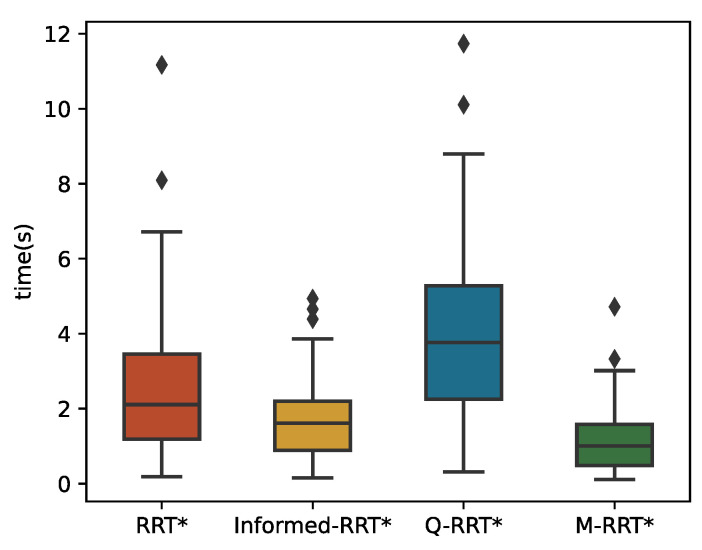
*T*_5%_ in the cluttered environment.

The 100 simulation results of *t_init_* and *T*_5%_ are described by boxplots, respectively. Table 3 counts the specific numerical values of the simulation results. As shown in Figure 13, in the cluttered environment, the median of M-RRT* is still the smallest, but the median of RRT* is larger than that of Informed-RRT*. Therefore, M-RRT* can obtain the initial solution earlier, while the other two algorithms are not stable. Q-RRT* still takes the longest time to find the initial path. As can be seen from Figure 14, although the efficiency of Informed-RRT* searching for the optimal solution has been improved compared to RRT*, M-RRT* is the most effective algorithm. Q-RRT* can continuously search ancestor vertices to optimize paths, but these paths often collide in the cluttered environments. Thus, Q-RRT* wastes a lot of time trying to change the structure of the tree instead, and it performs the worst in this environment. According to the running results of two performance indicators, M-RRT* has an outstanding performance in the cluttered environment.

### 4.4. Maze

**Figure 15:**
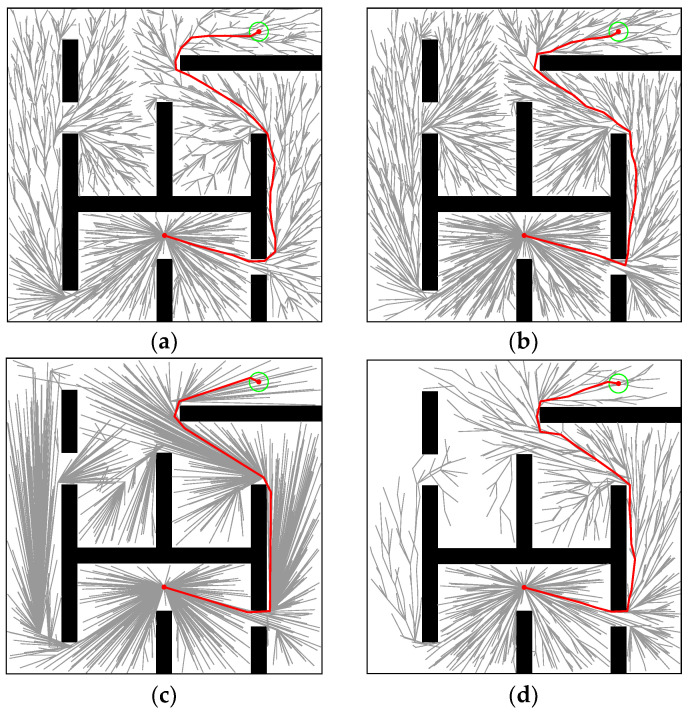
Performance of the four algorithms in the Maze. (**a**) RRT*. (**b**) Informed-RRT*. (**c**) Q-RRT*. (**d**) M-RRT*. (Red line: approximate optimal path, green circle: goal region.).

The maze is shown in Figure 8c. In Figure 15, the paths generated by RRT* (*t_init_* = 0.2163, *T*_5%_ = 4.1688), Informed-RRT* (*t_init_* = 0.375, *T*_5%_ = 14.4523), Q-RRT* (*t_init_* = 0.8908, *T*_5%_ = 1.5375), and M-RRT* (*t_init_* = 0.0647, *T*_5%_ = 1.3433) are shown. In the maze, *l_optimal_* = 138.6089. It can be seen that Informed-RRT* always performed global sampling like RRT*. Thus, Informed-RRT* has no advantage in this environment. Q-RRT* optimized paths more efficiently in the maze. On the contrary, due to the two vertex acceptance criteria proposed in this paper, M-RRT* rejected a lot of inferior vertices and obtained the solution with fewer vertices.

**Figure 16:**
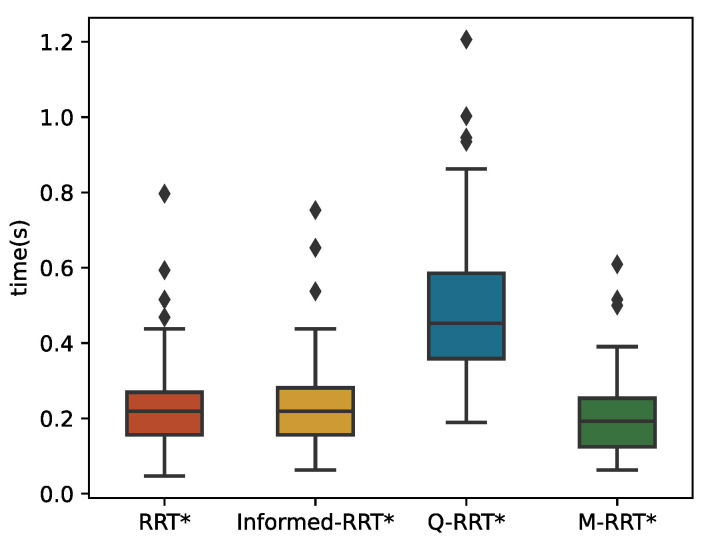
*t_init_* in the Maze.

**Figure 17:**
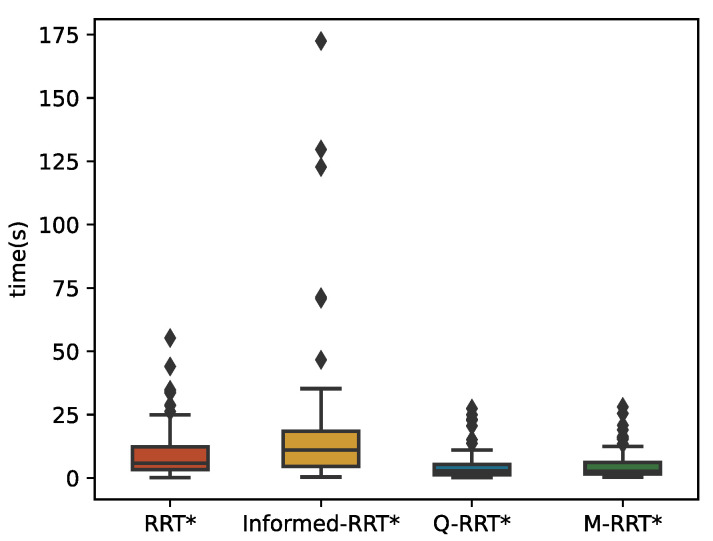
*T*_5%_ in the Maze.

Figure 16 and Figure 17 show 100 simulation results of *t_init_* and *T*_5%_. Table 4 counts the specific numerical values of the simulation results. Since maze-type environments tend to trap the algorithm in a local optimum, M-RRT* requires more extensive sampling when estimating the initial solution. It can be seen from Figure 16 that Q-RRT* takes the longest time, and although the medians of the other three algorithms are relatively close, the time needed for M-RRT* to obtain the initial solution is slightly less. As shown in Figure 17, M-RRT* obviously has the fastest convergence rate, while Informed-RRT* has the worst performance. The region of the hyper-ellipsoid set by Informed-RRT* in searching for the optimal solution in this environment is larger than the state space, which causes it to converge even slower than RRT*. The convergence speed of Q-RRT* is also very fast, so this paper indirectly proves that when Q-RRT* has enough space to search for ancestor vertices without collision, its efficiency will be greatly improved. Although RRT* performs worse, the minimum values of its two indicators are the lowest. This is because, in one of the experiments on RRT*, the random vertices of the algorithm found the path very quickly, which is accidental. By combining Figure 16 and Figure 17 and Table 4, it can be seen that M-RRT* performs significantly better in the maze.

## 5. Conclusions

There has been an increase in research on sampling-based motion planning algorithms in recent years. RRT* is a commonly used optimal algorithm, but this method has the problems of a low search efficiency and a slow convergence speed. M-RRT* is proposed to solve the motion planning problem of mobile robots. First, this research proposes an asymptotic vertex acceptance criterion in the initial path estimation phase of M-RRT*, which can effectively reduce the time of finding the initial path and make the algorithm start searching for the optimal path earlier. Secondly, this research proposes a nonlinear dynamic vertices acceptance criterion in the optimal path search phase of M-RRT*. This criterion preferentially accepts vertices that may improve the current path so as to rapidly converge to the optimal path.

Although M-RRT* is a promising algorithm, it can only rely on global sampling to jump out of the local optimum when finding initial solutions in some special environments. Moreover, this paper mainly studies static motion planning, but the actual environment is dynamic, so we should take into account the real-time nature of motion planning in a further work. Since the motion planning of the manipulators has complex constraints, a small number of path points can reduce its calculation. We apply the algorithm proposed in this paper to the motion planning of the manipulator as the next research content.

## Figures and Tables

**Table 1:** System and resource characteristics.

Simulation Environment	System	CPU	GPU	RAM
Python 3.9	Windows 10 Professional edition	Intel(R) Core(TM) I5-110400F	NVIDIA GeForce GTX 1050	16 G

**Table 2:** *t_init_* and *T*_5%_ of four algorithms in the regular environment.

Algorithm		Mean	Median	Min	Max
RRT*	*t_init_*	0.1273	0.1093	0.031243	0.4374
	*T*_5%_	2.1116	0.9294	0.031243	13.5426
Informed-RRT*	*t_init_*	0.1385	0.1249	0.031241	0.453
	*T*_5%_	0.488	0.4061	0.031241	3.1242
Q-RRT*	*t_init_*	0.2739	0.2233	0.0678	0.9514
	*T*_5%_	1.8275	0.8547	0.0678	14.1172
M-RRT*	*t_init_*	0.0799	0.0624	0.0156	0.2499
	*T*_5%_	0.3159	0.1952	0.0156	1.2653

**Table 3:** *t_init_* and *T*_5%_ of four algorithms in the cluttered environment.

Algorithm		Mean	Median	Min	Max
RRT*	*t_init_*	0.3999	0.3514	0.1405	1.1716
	*T*_5%_	2.5054	2.1088	0.1874	11.1693
Informed-RRT*	*t_init_*	0.3716	0.3124	0.1093497	1.3278
	*T*_5%_	1.6417	1.6089	0.1562	4.9363
Q-RRT*	*t_init_*	1.0989	0.9808	0.3127	2.3687
	*T*_5%_	4.0272	3.7631	0.3127	11.7356
M-RRT*	*t_init_*	0.3002	0.2811	0.1093492	0.8123
	*T*_5%_	1.1838	1.0075	0.1093492	4.7174

**Table 4:** *t_init_* and *T*_5%_ of four algorithms in the Maze.

Algorithm		Mean	Median	Min	Max
RRT*	*t_init_*	0.2365	0.2186	0.0468	0.7966
	*T*_5%_	9.445	5.8267	0.1874	55.2755
Informed-RRT*	*t_init_*	0.2375	0.2030	0.06248	0.753
	*T*_5%_	17.0028	11.0589	0.406157	172.453
Q-RRT*	*t_init_*	0.4876	0.4523	0.1894	1.206
	*T*_5%_	4.5829	2.7853	0.2269	27.4085
M-RRT*	*t_init_*	0.2062	0.1921	0.06247	0.6092
	*T*_5%_	5.091	2.7571	0.406155	28.0715

## Data Availability

Not applicable.
